# Missed and mismanaged: severe consequences of Morel Lavallee lesions—a case series

**DOI:** 10.1093/jscr/rjaf731

**Published:** 2025-09-14

**Authors:** Hamad AlJuwied, Dalal AlNajadah, Zahraa Mostafa, Ali Ashraf, Ali Jarragh, Ali Lari

**Affiliations:** Department of Orthopedic Surgery, AlRazi Orthopedic Hospital, Kuwait City, Kuwait; Department of Orthopedic Surgery, Jaber Al Ahmed Hospital, Kuwait City, Kuwait; Royal College of Surgeons Ireland, Dublin 2, Ireland; Department of Surgery, Jaber Al Ahmed Hospital, Kuwait City, Kuwait; Department of Surgery, Kuwait University, Kuwait City, Kuwait; Department of Orthopedic Surgery, AlRazi Orthopedic Hospital, Kuwait City, Kuwait

**Keywords:** Morel Lavallee lesion, closed degloving, delayed diagnosis, skin graft, complications

## Abstract

Morel-Lavallée lesions (MLLs) are rare soft tissue injuries that, when missed or mismanaged, can lead to serious complications. This retrospective case series analyzed seven male patients (aged 26–64) who sustained MLLs from high-velocity trauma. Initial management, often limited to aspiration, failed in most cases, leading to delayed surgical intervention (mean 25.7 days post-injury) and an average of 2.6 surgeries per patient. Complications included skin necrosis (n = 5), grafting (n = 4), and infection in all cases, most commonly Staphylococcus aureus. One patient died from cardiogenic and septic shock. These findings highlight that delayed recognition and conservative treatment often result in prolonged morbidity, whereas early surgical debridement and proper closure may significantly improve outcomes.

## Introduction

Morel-Lavallée lesions (MLLs) are uncommon soft tissue injuries with significant implications for patient outcomes. Despite their rarity, MLLs present challenges in both diagnosis and management [1]. MLLs are closed degloving injuries caused by high-velocity shear forces that separate skin and subcutaneous tissue from fascia, creating a fluid-filled space [2]. Most MLL cases are associated with fractures in various anatomical locations including proximal femoral and pelvic injuries [3, 4]. MLLs are primarily diagnosed through clinical evaluation, with imaging modalities used to investigate size, presence of a pseudocapsule and location [5]. An ultrasound may be more useful in identifying and localizing fluid collection [6]. Magnetic Resonance imaging remains the gold standard modality for MLL [6].

The management of MLLs requires a comprehensive understanding of their etiology, pathophysiology, clinical presentation, and treatment options [5]. There is no clear consensus on optimal MLL management, but the primary goal is fluid evacuation, aspiration for small lesions and surgery for larger or delayed cases [5]. Complications such as skin necrosis, infection, and recurrence can arise, underscoring the importance of early recognition and appropriate management [5, 6]. Early recognition and prompt management is key in limiting morbidity [3]. This study highlights the severity of complications in a series of missed and mismanaged MLLs.

## Methods

This retrospective case series analyzed prospectively collected data on eight patients with missed or mismanaged MLLs treated at a single tertiary orthopedic center between 2019 and 2023 (Tables 1 and 2). All patients were followed for at least 18 months. Data were obtained from institutional databases and medical records, with written consent provided by patients. Conservative treatment failure was defined by persistent lesion size, skin necrosis, infection, or fluid re-accumulation after aspiration. Surgical interventions, time to surgery, complications, negative pressure wound therapy (NPWT)/grafting, and microbial findings were recorded.

**Table 1:** Patient and injury characteristics.

**Case number**	**Age**	**Gender**	**Mechanism of injury**	**Associated injuries**	**Location of lesion**	**Organisms**
1	31	Male	Forklift/construction vehicular injury	Open book pelvic fracture, ankle ligamentous injury, pneumothorax, Open groin wound flap extending to groin	Thigh	MRSA, *K. Pneumoniae*
2	41	Male	RTA	Contralateral multiligamentous knee injury	Thigh	*P. Aeruginosa*, *K. Pneumoniae*
3	28	Male	RTA	Open book pelvic fracture	Peripelvic	*P. Aeruginosa*
4	37	Male	RTA	Acetabulum posterior column, wall	Peripelvic	*S. Aureus*
5	39	Male	Pedestrian RTA	Subtrochanteric femoral fracture	Peripelvic	*S. Aureus*, *B. Fragilis*
6	43	Male	Pedestrian RTA	Ipsilateral femoral fracture	Knee	*S. Aureus*, *P. Mirabilis*
7	26	Male	RTA	Acetabulum posterior column	Peripelvic	*S. Aureus*
8	64	Male	Pedestrian RTA	Avulsed heel pad	Leg/ Ankle	*S. Aureus*, *P. Aeruginosa*

**Table 2:** Clinical outcomes and complications of the included participants.

**Case number**	**Failed treatment received**	**Complications**	**Time from injury to surgery (for lesion) (Days)**	**Surgical treatment received**	**NPWT**	**Graft**	**Number of procedures**
1	Wound closure, irrigation, 2 redivac drains	Entire thigh circumferentially, knee, part of groin necrosis. Muscle necrosis requiring excision. Septic shock, intubated, dual vasopressors	16	Multiple debridements and skin grafts		Massive split thickness	7
2	2–3 aspiration attempts	Skin necrosis 15 × 4 cm requiring graft, followed by partial graft necrosis treated conservatively	26	Surgical debridement, layered closure of dead space, excision of necrotic skin	NPWT	Split thickness	3
3	Aspiration	Skin necrosis 2 × 3 cm, secondary intention	16	Debridement, irrigation, secondary wound closure	NPWT	None	1
4	Aspiration		22	Debridement, irrigation, primary wound closure		None	2
5	Two aspiration attempts		52	Debridement, irrigation, primary wound closure		None	1
6	Two aspiration attempts	Skin necrosis 5 × 2 cm	20	Evacuation, debridement, primary wound closure		Split thickness	2
7	Irrigation and closure of skin	Skin necrosis 4 × 5 cm, requiring graft	28	Debridement	NPWT	Split thickness	2
8	Wound closure	Skin necrosis circumferential bilateral legs Death due to cardiogenic & septic shock	21	Debridement		Split thickness	3
Mean			25.1				2.6

## Results

### Patient characteristics

All eight patients were male, aged 26–64 (mean: 38.6), with an average follow-up of 20 months. Injuries stemmed from high-velocity trauma: road traffic accidents (RTAs) (n = 4), pedestrian RTAs (n = 3), and a construction accident (n = 1). Associated fractures included the femur (n = 2), acetabulum (n = 2), pelvis (n = 2), heel (n = 1), and knee (n = 1) (Table 1).

MLLs were located in the peri-pelvic region (n = 4), thigh (n = 2), below knee and ankles (n = 1), and knee (n = 1). Initial conservative measures, aspiration (n = 5), minor debridement or closure (n = 3) were unsuccessful. All patients required surgery, averaging 2.6 procedures, with surgery occurring on average 25.1 days post-injury (Table 2).

Complications included delayed skin necrosis (n = 6), necessitating split-thickness grafts (n = 5) and NPWT (n = 3) (Table 2). All lesions had positive tissue cultures, predominantly Staphylococcus aureus (n = 6), followed by Pseudomonas aeruginosa (n = 3) (Table 1). Most lesions presented as fluctuant swellings without initial skin degloving, though necrosis developed later in three of these cases. Six patients underwent multiple surgeries for debridement and reconstruction (Table 2). One patient aged 64 with severe baseline cardiac failure died following the third debridement and skin grafting of a single leg. The patient was admitted to the intensive care unit, requiring inotropic support and subsequently died of cardiac arrest after 4-week hospital stay.

### Case highlight 1: Severe mismanaged MLL with septic shock

A 31-year-old male sustained a severe MLL following a construction accident, presenting with an open-book pelvic fracture, pneumothorax, and a large open groin wound extending to the knee. Initial management at a general hospital involved percutaneous sacroiliac screw fixation, pelvic external fixation, and irrigation with debridement. However, the debridement was inadequate, lacking layered closure of dead space and aggressive necrotic tissue removal. Two drains were placed, persistently draining serosanguinous fluid.

**Figure 1:**
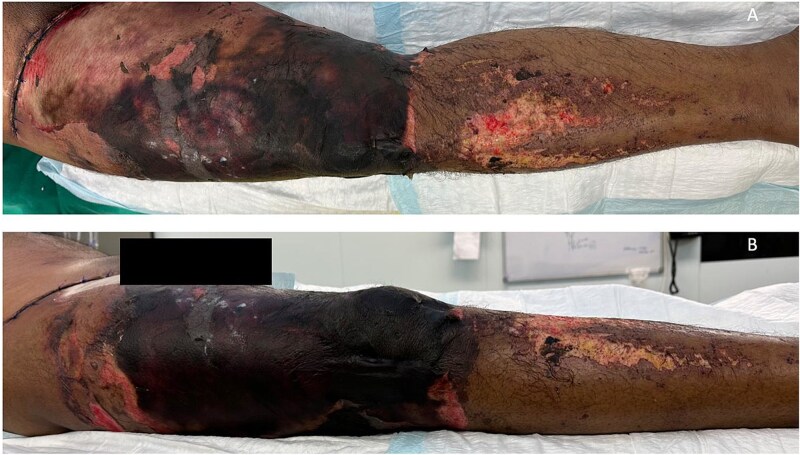
Case highlight 1—Severe circumferential necrosis of the thigh.

**Figure 2:**
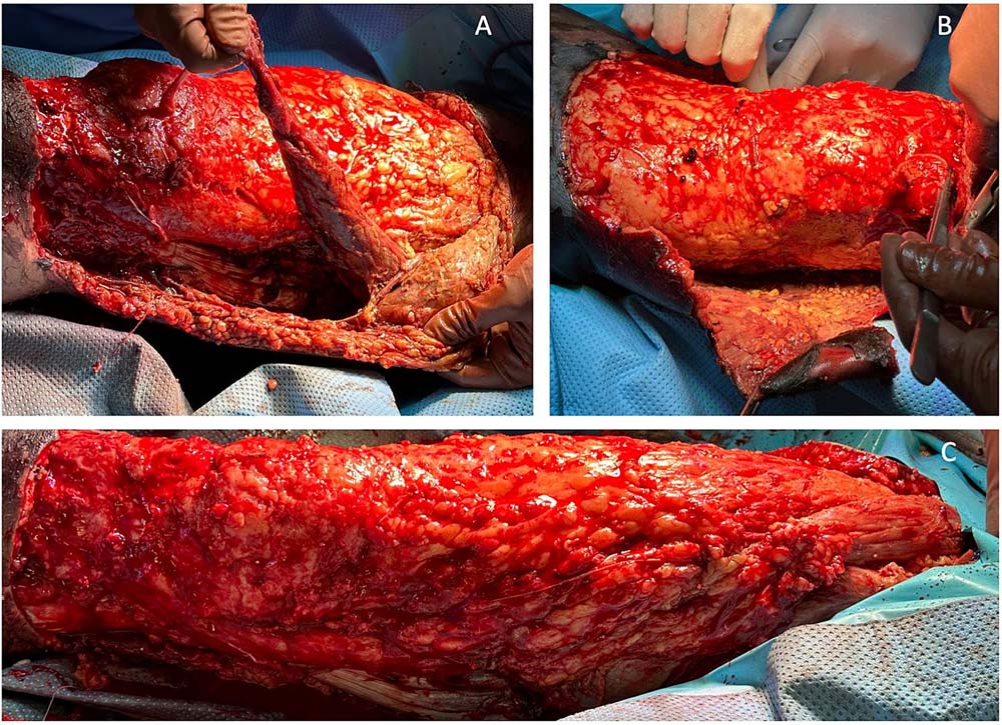
Case highlight 1—Intraoperative image showing debridement, skin, and muscle excision.

The patient’s condition deteriorated rapidly, developing extensive gangrene of the right thigh and part of the knee (Fig. 1). He became hemodynamically unstable with septic shock, requiring intubation and dual vasopressor support. Emergency surgical intervention involved extensive debridement and excision of necrotic tissue and muscle (Fig. 2).

**Figure 3:**
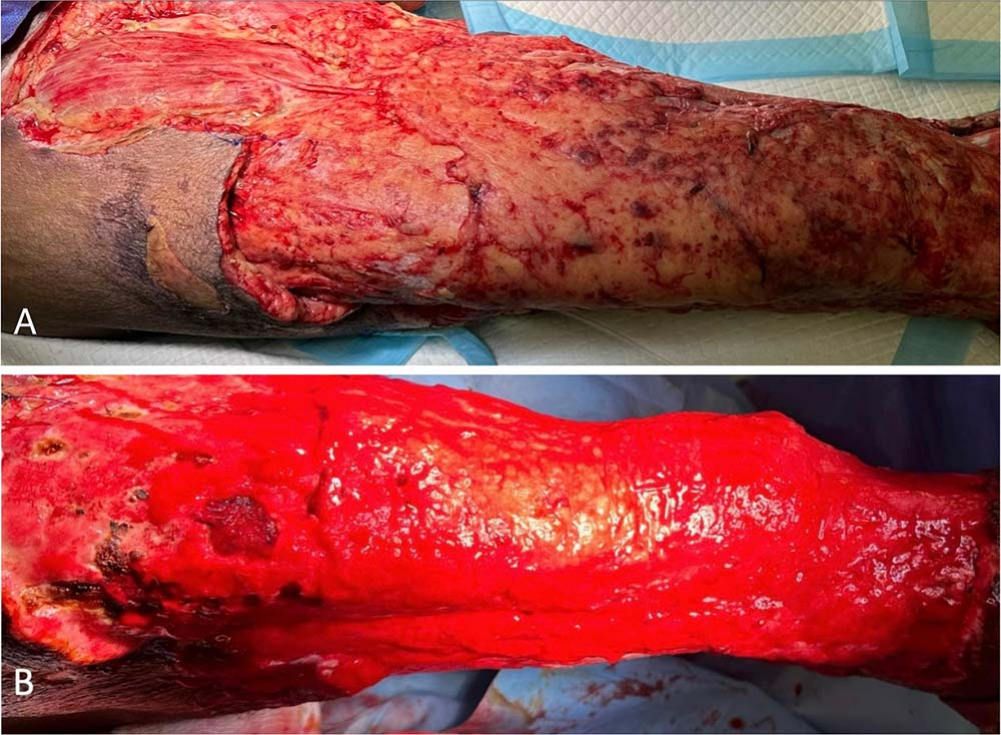
Case highlight 1—Intraoperative image of third session of debridement ⁓1 month after initial trauma.

**Figure 4:**
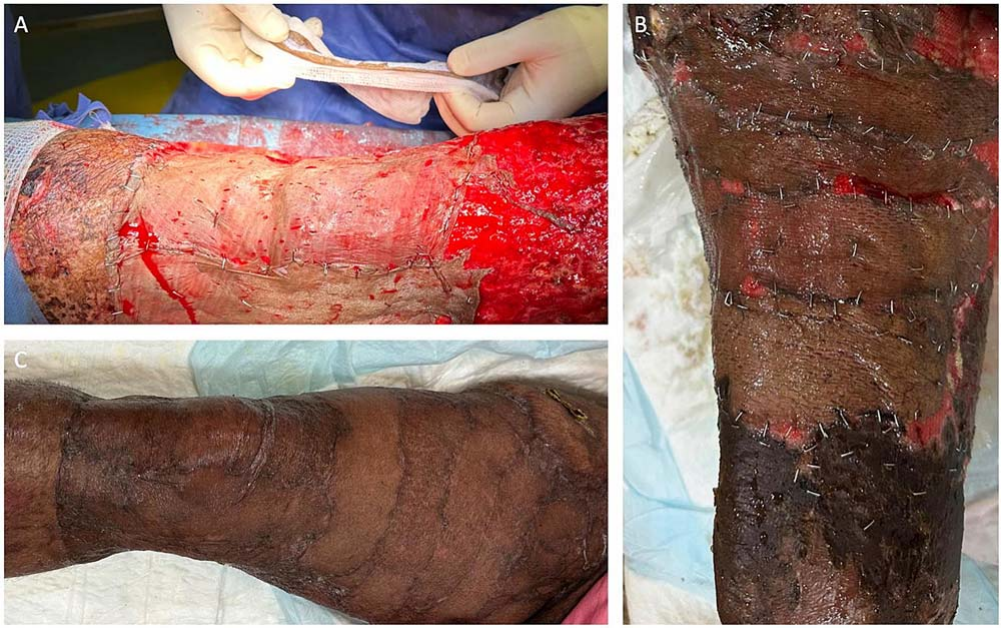
Intraoperative and postoperative images from case 1 following multiple split thickness grafts.

Postoperatively, he received targeted IV antibiotics, intensive physiotherapy to prevent contractures, and dietary optimization with increased protein intake. Daily hypertonic saline dressings were applied, and he underwent multiple split-thickness skin grafts to manage the large tissue defect (Figs 3 and 4). His total hospital stay was 95 days. At 18-month follow-up, he showed adequate healing and improved mobility.

### Case highlight 2: Large MLL with failed multiple aspiration attempts

**Figure 5:**
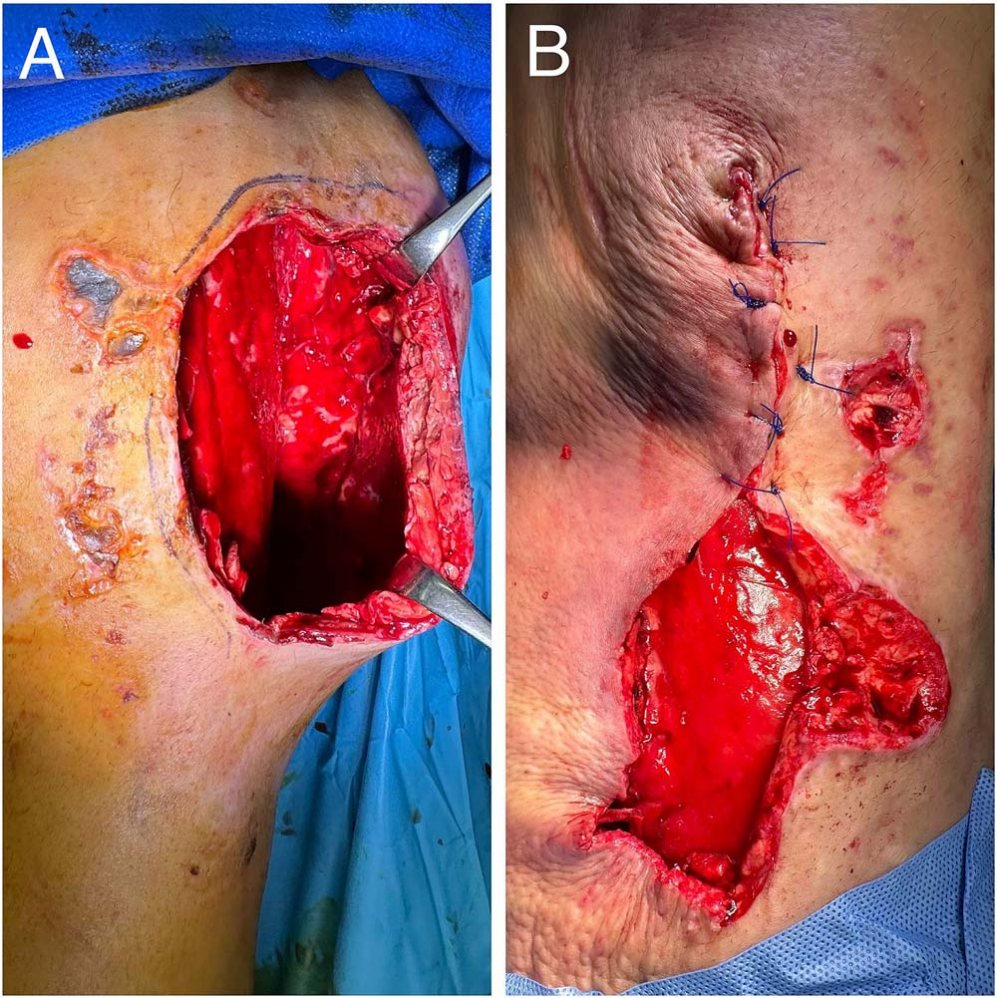
Intraoperative images from case 2 showing large dead space and subsequent layered closure.

**Figure 6:**
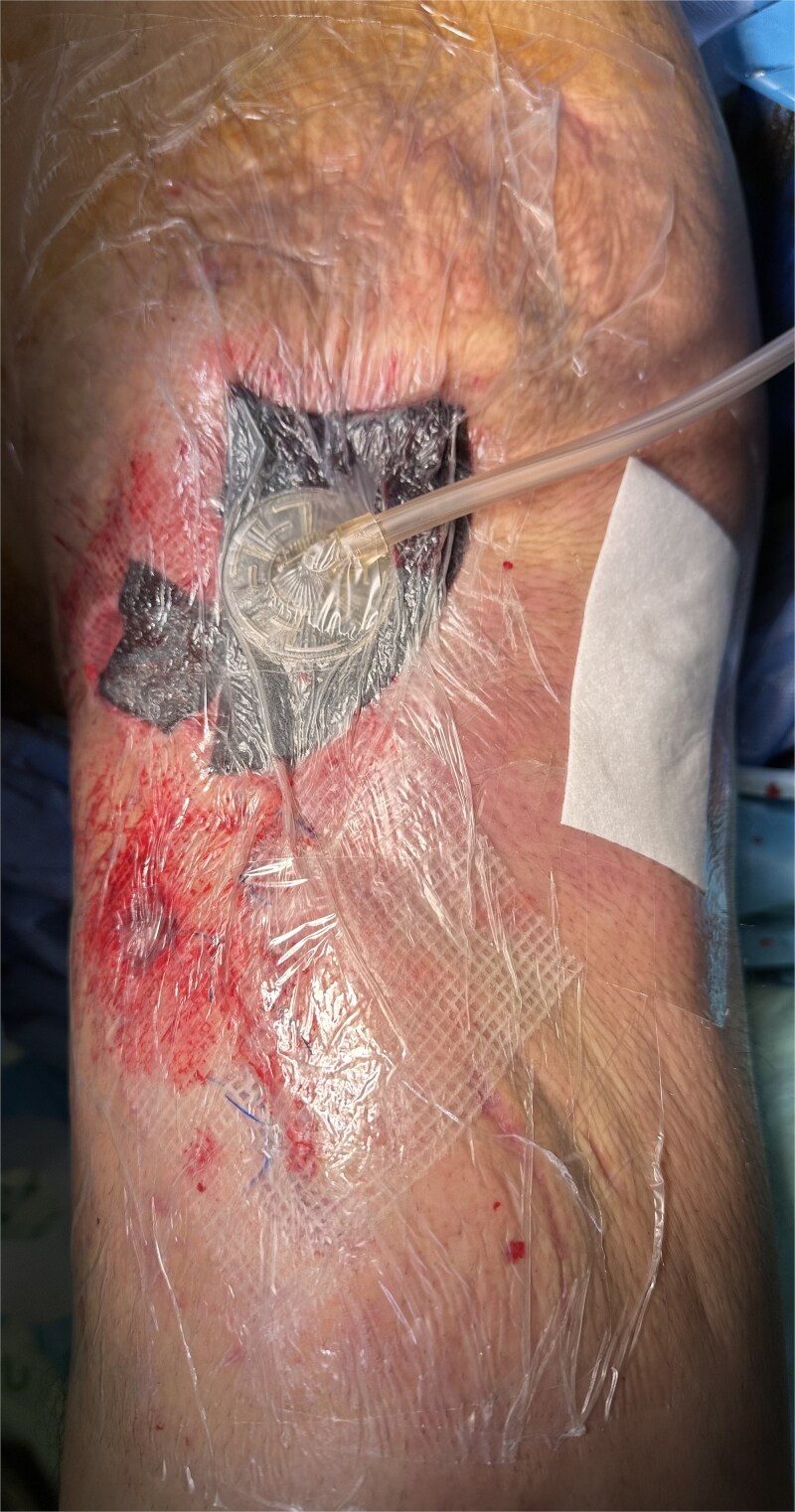
Intraoperative image from case 2 showing negative pressure wound therapy.

**Figure 7:**
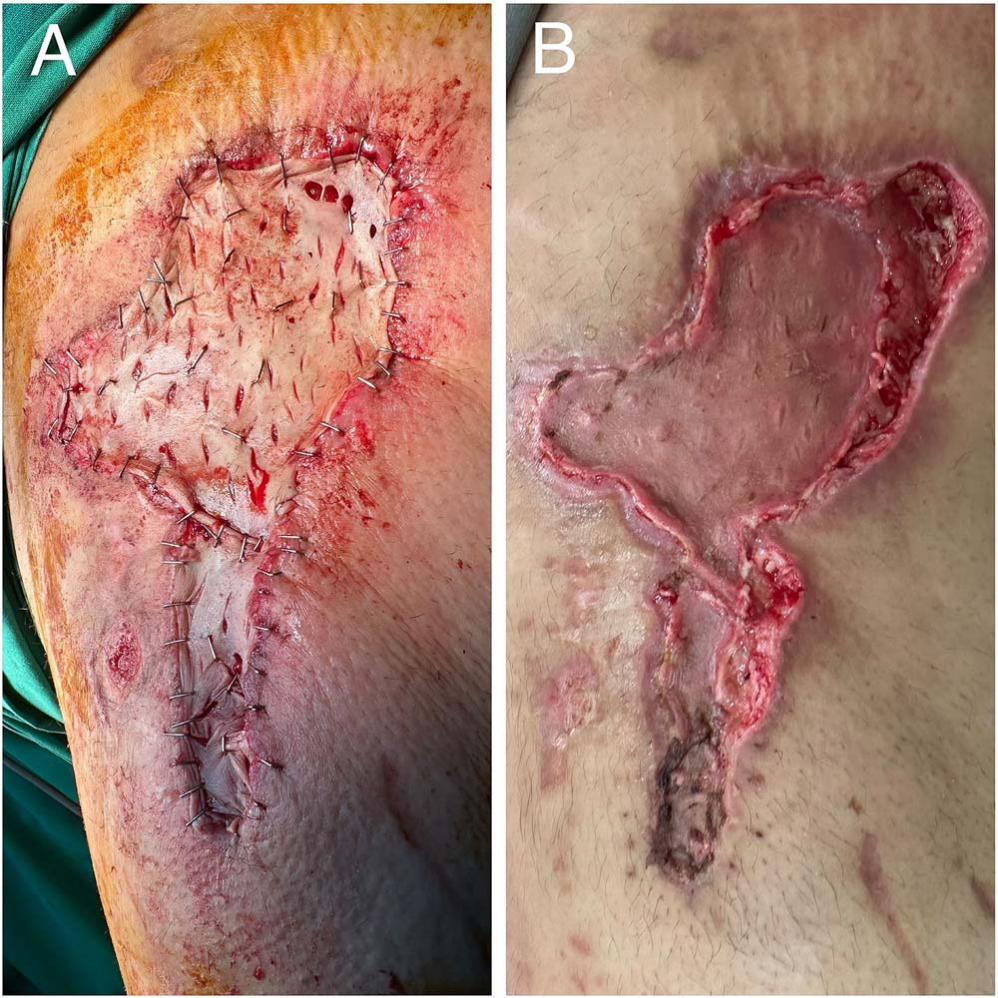
Images from case 2. (A) Intraoperative image showing split thickness grafting. (B) Postoperative partial graft necrosis.

**Figure 8:**
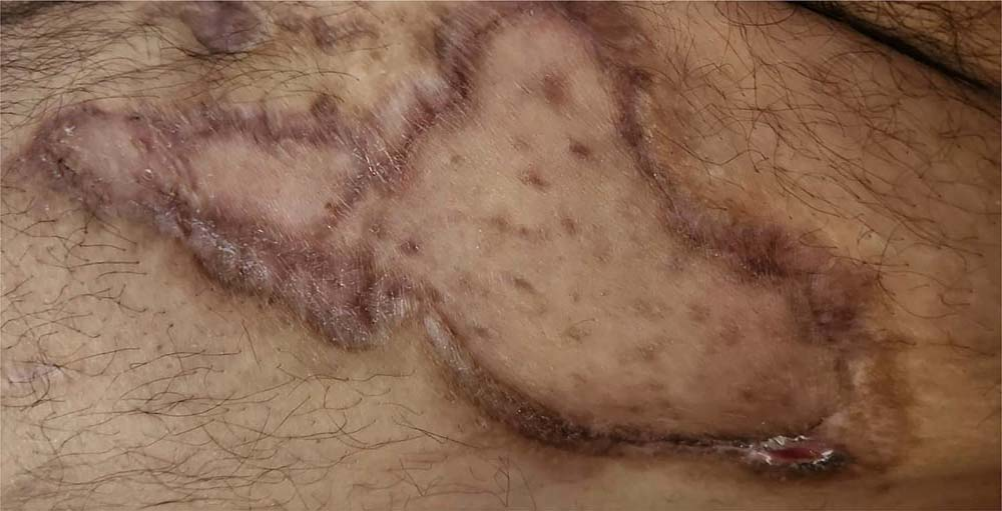
Image from case 2 showing full healing.

A 41-year-old male involved in an RTA presented with a multi-ligamentous knee injury and a necrotic skin patch on the anterolateral thigh. Initial multiple aspirations at a general hospital were ineffective, and upon referral, a 15 × 4 cm necrotic patch was identified. Immediate debridement and cultures confirmed Pseudomonas and Klebsiella infection. Initial surgery involved debridement with layered closure and negative pressure wound therapy (Figs 5 and 6). The patient underwent three procedures and showed good healing at 12 months, although partial graft necrosis later developed that healed with secondary intention (Figs 7 and 8). Overall satisfaction was affected; hospital stay extended and multiple procedures were unnecessarily required.

## Discussion

The findings from this series highlight the considerable morbidity associated with missed or delayed diagnosis and treatment of MLLs, including infection, skin necrosis, prolonged hospital stays, and multiple surgeries required to manage the lesions and their associated complications. In our series, aspiration was often initially utilized without success, necessitating subsequent open debridement. All patients had positive tissue cultures for infectious organisms, and six cases developed substantial skin necrosis as a complication. Most patients required multiple surgical interventions (6 of 8) and had considerably long hospital stays.

Delayed diagnosis is commonly cited in the literature. In our cohort, missed MLL were primarily attributed to distracting injuries. Nicolas *et al*. noted that MLLs were missed in one-third of cases, with some lesions taking weeks to months to diagnose [5, 7]. Kottmeier *et al.* found that 44% of MLLs in their study of 16 cases were initially overlooked [7]. The time from injury to diagnosis and intervention varies across studies. Rodríguez-Roiz *et al.* described nine MLL cases with an average time from injury to diagnosis of 11.9 days (range, 8–17 days), with percutaneous drainage performed on average 23.4 days post-diagnosis (range, 14–35 days) [8]. Tseng *et al.* reported an average time from injury to debridement of 13.1 days [9]. Comparatively, our mean time to surgical intervention was 25.1 days.

**Figure 9:**
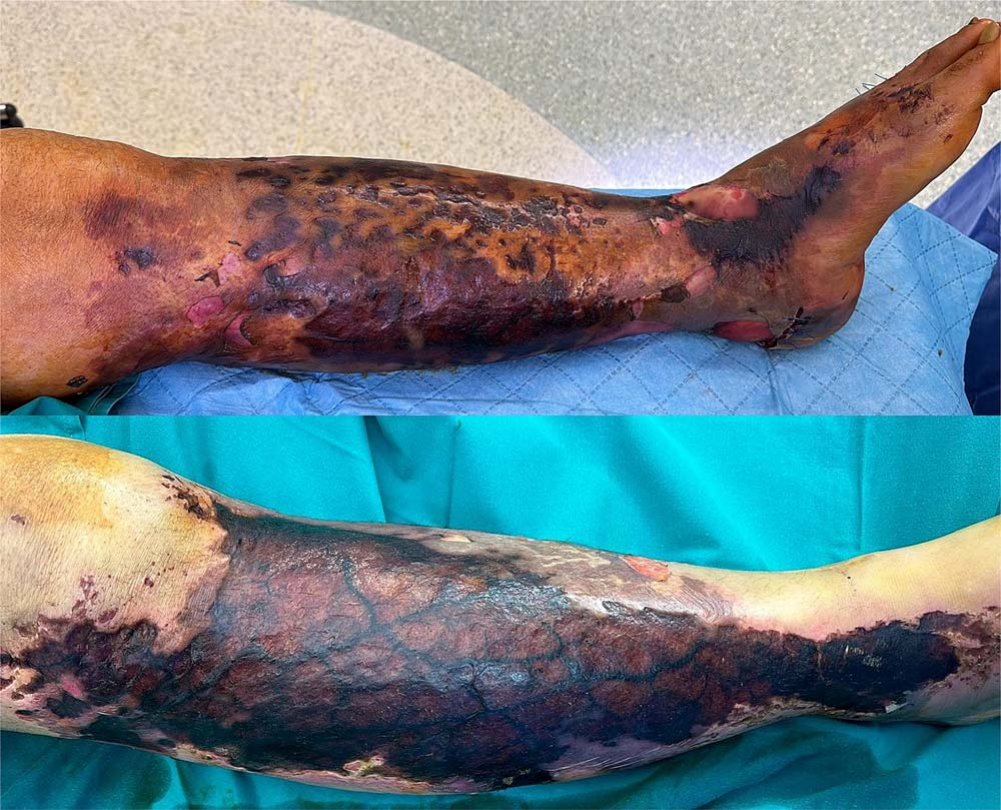
Image of case 8—64 year old with bilateral necrosis.

The underlying reason for missing these injuries is likely multifaceted and may initially stem from the distracting injuries that often accompany MLLs. Another potential cause is misdiagnosing the injury as a minor contusion or simple hematoma. This particular reason may be due to a lack of familiarity with the healthcare providers. As such, clinicians should maintain a high index of suspicion for MLL in patients with polytrauma resulting from high-velocity impacts [2] (Fig. 9). We recommend the secondary survey includes a thorough examination of common locations for MLLs and manages them accordingly. In addition, any doubt should be supplemented by advanced imaging ranging from ultrasound to magnetic resonance imaging.

Early clinical manifestations include swelling and tenderness, seroma formation characterized by persistent swelling, ecchymosis, and skin discoloration [3, 5, 6, 10]. Some patients may be asymptomatic, which complicates diagnosis, as noted by Jalota *et al*., who reported a missed diagnosis rate of up to 33% at initial assessment [3]. Fluctuance at the lesion site is a prominent feature, although paraesthesia, which can occur due to injury to the neurovascular bundle, was not observed in our patients [6, 11].

While MLLs are primarily diagnosed clinically, imaging modalities such as magnetic resonance imaging (MRI), ultrasound, and computed tomography (CT) may aid in assessment [4, 12, 13]. The role of plain radiography is limited but may show a delineated soft tissue mass [6]. CT can show fluid–fluid levels as a result of blood/lymph accumulation as well as fat or tissue debris [6]. This may also be associated with an encircling capsule [6]. The value of MRI is that it can clearly visualize the margins of the cystic lesions, the fascial planes and cystic components. Shen *et al.* suggested that MRI findings might guide treatment options in peri-pelvic MLLs [14].

The approach to treating MLLs is variable in current literature [5]. The standard management of large, missed, and infected peri-trochanteric Morel-Lavallée lesions involves surgical debridement with layered dead space closure, followed by appropriate wound coverage. Aspiration alone is rarely effective, as recurrence is common and may lead to infection and delayed skin necrosis. Our series underscores this diagnostic and therapeutic pitfall. Early, definitive debridement with proper closure likely would have prevented the progression to extensive necrosis, aligning with findings from similar studies in the literature [8, 15], suggesting that conservative measures should be reserved for small, well defined and acute lesions that are unlikely to be colonized.

Compressive therapy may result in recurrence if used alone, but can be an effective adjunct to other treatment options such as aspiration and surgical interventions, especially in minor lesions [13, 14, 16]. A retrospective study including 25 patients yielded positive outcomes with compression bandaging, however recovery can take relatively longer compared to more invasive options [16]. Our cases, in which aspiration failed, align with literature reporting high recurrence rates with percutaneous aspiration [14, 16]. Failed aspiration usually occurs due to the large lesion size which means more aggressive intervention is needed [14, 16]. Sclerodesis, involving the injection of a sclerosing agent into the lesion, prevents recurrence by forming fibrous scar tissue and is effective for lesions up to 400 ml [10, 17]. However, it carries risks such as infection, skin hardening, and postoperative pain [14, 18].

Percutaneous drainage has been reported to be effective for small lesions (<50 ml) [8, 16]. This technique was established by Tseng and Tornetta with the aim of preserving the subdermal arterial plexus [9]. A case report by Zhong *et al.* assessed the role of percutaneous drainage in eight patients with a 1-week delayed diagnosis and demonstrated excellent efficacy [19]. However, large or complicated lesions, such as those in our series, require debridement, particularly due to the delay in diagnosis, severity of injury and bacterial associated necrosis [19]. Video-assisted endoscopic debridement with percutaneous cutaneo-fascial suturing is a novel, less invasive alternative to open surgery [20]. The current standard for managing large MLLs with substantial skin necrosis is initial debridement followed by appropriate wound coverage. NPWT is beneficial for managing infected lesions, a common complication associated with MLLs [14, 16]. Suzuki *et al.* reported an 8.4-fold increase in the risk of infection with MLLs [21]. The increased infection rate in our cases may be attributed to delayed diagnosis and surgical management. Takahara *et al.* found that positive cultures were more common in cases where surgical management was delayed beyond three days after trauma. Nicolas *et al.* and Takahara *et al.* reported cases of infected MLLs requiring prolonged hospital stays and multiple surgical interventions, similar to our cases [15].

The presence of persistent complications necessitating multiple surgeries prolongs recovery and hospital stays, negatively impacting patient satisfaction [22]. Return to work is another factor affecting satisfaction; Rodríguez-Roiz *et al.* reported earlier returns to work with surgical interventions compared to nonoperative management [8]. Split-thickness grafting can also have unfavorable cosmetic outcomes, further impacting patient satisfaction [23]. Effective communication and setting realistic expectations pre-surgery are crucial for improving outcomes and patient satisfaction [24, 25].

This study has several limitations. Firstly, as a retrospective and purely observational study, it is inherently subject to biases associated with such designs. Additionally, the inclusion criteria were specifically designed to highlight poor outcomes related to delayed diagnosis and mismanagement of MLLs. While this approach aligns with the study’s objective, it may have introduced a selection bias, potentially overemphasizing less favorable outcomes.

## Conclusion

Prompt diagnosis and appropriate management of MLLs are essential to prevent complications such as chronic lesions, skin necrosis, and infection. Multiple aspirations of large, delayed lesions are often ineffective, making early debridement crucial in reducing morbidity, shortening hospital stays, and minimizing the number of required procedures.

## Author declaration

We, the authors of this submission confirm that we have not published the same or a very similar study with the same or very similar results and major conclusions in any other journals. These include English or non-English language journals and journals that are indexed or not indexed in PubMed, regardless of different words being used in the article titles, introduction, and discussion.

## References

[1] Nair AV, Nazar P, Sekhar R, et al. Morel-Lavallée lesion: a closed degloving injury that requires real attention. Indian J Radiol Imaging 2014;24:288–90. 10.4103/0971-3026.137053 25114393 PMC4126145

[2] Molina BJ, Ghazoul EN, Janis JE. Practical review of the comprehensive management of Morel-Lavallée lesions. Plastic Reconstruct Surg Global Open 2021;9:e3850. 10.1097/GOX.0000000000003850 PMC8500644 34646720

[3] Jalota L, Ukaigwe A, Jain S. Diagnosis and management of closed internal degloving injuries: the Morel-Lavallée lesion. J Emerg Med 2015;49:e1–4. 10.1016/j.jemermed.2014.12.084 25843923

[4] Hu M, Chen J, Ma L, et al. The treatment of a Morel-Lavallée lesion of the thigh with incision and drainage along with tissue debridement and a surgically placed drain: a case report and literature review. Front Surg 2023;9:1071421. 10.3389/fsurg.2022.1071421 36684196 PMC9857385

[5] Diviti S, Gupta N, Hooda K, et al. Morel-Lavallee lesions-review of pathophysiology, clinical findings, imaging findings and management. J Clin Diagn Res 2017;11:TE01–4. 10.7860/JCDR/2017/25479.9689 PMC5449878 28571232

[6] Sohail AH, Liaquat MT, Sohail MS, et al. Morel-Lavallée lesion in a 35-year female. J Coll Physicians Surg Pak 2021;31:342–5. 10.29271/jcpsp.2021.03.342 33775030

[7] Kottmeier SA, Wilson SC, Born CT, et al. Surgical management of soft tissue lesions associated with pelvic ring injury. Clin Orthop Relat Res 1996;329:46–53. 10.1097/00003086-199608000-00007 8769435

[8] Rodríguez-Roiz JM, Burillo JM, Díaz JSS. Morel-Lavallee lesions. Size matters? Treatment and time of disability. Injury 2023;54:150–3. 10.1016/j.injury.2022.10.023 36328805

[9] Tseng S, Tornetta P. 3^rd^ percutaneous management of Morel-Lavallee lesions. J Bone Joint Surg Am 2006;88:92–6. 10.2106/JBJS.E.00021 16391253

[10] Singh R, Rymer B, Youssef B, et al. The Morel-Lavallée lesion and its management: a review of the literature. J Orthop 2018;15:917–21. 10.1016/j.jor.2018.08.032 30190632 PMC6126206

[11] Gelber J, Sher W. Morel-Lavallée lesion diagnosed by point-of-care ultrasound: a case report and review of treatment strategies. J Emerg Med 2023;64:74–6. 10.1016/j.jemermed.2022.10.021 36642674

[12] Puig J, Pelaez I, Baños J, et al. Long-standing Morel-Lavallée lesion in the proximal thigh: ultrasound and MR findings with surgical and histopathological correlation. Australas Radiol 2006;50:594–7. 10.1111/j.1440-1673.2006.01640.x 17107533

[13] Hudson DA, Knottenbelt JD, Krige JE. Closed degloving injuries: results following conservative surgery. Plast Reconstr Surg 1992;89:853–5. 10.1097/00006534-199205000-00013 1561257

[14] Shen C, Peng JP, Chen XD. Efficacy of treatment in peri-pelvic Morel-Lavallee lesion: a systematic review of the literature. Arch Orthop Trauma Surg 2013;133:635–40. 10.1007/s00402-013-1703-z 23443527

[15] Nicolas G, Abbas L, Prado A, et al. Case report: stage VI Morel-Lavallée lesion with a large challenging defect. Plastic Reconstruct Surg Global Open 2021;9:e3502. 10.1097/GOX.0000000000003502 PMC8081468 33936913

[16] Nickerson TP, Zielinski MD, Jenkins DH, et al. The Mayo Clinic experience with Morel-Lavallée lesions: establishment of a practice management guideline. J Trauma Acute Care Surg 2014;76:493–7. 10.1097/TA.0000000000000111 24458056

[17] Penaud A, Quignon R, Danin A, et al. Alcohol sclerodhesis: an innovative treatment for chronic Morel-Lavallée lesions. J Plast Reconstr Aesthet Surg 2011;64:e262–4. 10.1016/j.bjps.2011.06.012 21741333

[18] Scolaro JA, Chao T, Zamorano DP. The Morel-Lavallée lesion: diagnosis and management. J Am Acad Orthop Surg 2016;24:667–72. 10.5435/JAAOS-D-15-00181 27579812

[19] Zhong B, Zhang C, Luo CF. Percutaneous drainage of Morel-Lavallée lesions when the diagnosis is delayed. Can J Surg 2014;57:356–7. 10.1503/cjs.034413 25265112 PMC4183685

[20] Liu M, Liu L, Zhou X, et al. A novel surgical technique for treatment of Morel-Lavallée lesion: endoscopic debridement combined with percutaneous cutaneo-fascial suture. Injury 2018;49:1630–3. 10.1016/j.injury.2018.06.003 29891390

[21] Suzuki T, Morgan SJ, Smith WR, et al. Postoperative surgical site infection following acetabular fracture fixation. Injury 2010;41:396–9. 10.1016/j.injury.2009.11.005 20004894

[22] Alemu ME, Worku WZ, Berhie AY. Patient satisfaction and associated factors towards surgical service among patients undergoing surgery at referral hospitals in western Amhara regional State, Ethiopia. Heliyon 2023;9:e14266. 10.1016/j.heliyon.2023.e14266 36938460 PMC10015238

[23] Kanapathy M, Bystrzonowski N, Hachach-Haram N, et al. Lower donor site morbidity and higher patient satisfaction with epidermal grafting in comparison to split thickness skin grafting: a randomized controlled trial (EPIGRAAFT trial). J Plast Reconstr Aesthet Surg 2020;73:1556–64. 10.1016/j.bjps.2020.03.006 32532631

[24] Lari A, Alherz M, Hussain S, et al. The importance of scar cosmesis across the surgical specialties: factors, perceptions, and predispositions. Plast Reconstr Surg Glob Open 2022;10:e4219. 10.1097/GOX.0000000000004219 35356039 PMC8939916

[25] Lari A, Alherz M, Jarragh A. Dissociating advances in orthopaedic trauma management from the climbing patient expectations. Eur J Trauma Emerg Surg 2022;48:1487. 10.1007/s00068-021-01705-0 34028560

